# The inion response revisited: evidence for a possible cerebellar contribution to vestibular-evoked potentials produced by air-conducted sound stimulation

**DOI:** 10.1152/jn.00545.2016

**Published:** 2016-12-14

**Authors:** Neil P. M. Todd, Sendhil Govender, James G. Colebatch

**Affiliations:** ^1^Department of Psychology, University of Exeter, Exeter, United Kingdom; and; ^2^Prince of Wales Clinical School and Neuroscience Research Australia, University of New South Wales, Randwick, Sydney, Australia

**Keywords:** vestibular, VEMP, VsEPs, cerebellum

## Abstract

This study investigated the effect of eye gaze and head position on vestibular-evoked potentials (VsEPs). Head position would be expected to affect myogenic sources, and eye position is known to affect ocular myogenic responses (ocular vestibular-evoked myogenic potentials), whereas a neurogenic source should behave otherwise. Eleven healthy subjects were recruited, and VsEPs, using 72-channel EEG, were recorded at a fixed intensity above the vestibular threshold. Three eye gaze and three head positions were tested (−20°, 0°, and +20° to the horizontal). Short-latency potentials showed that in addition to the expected effect of gaze on infraocular (IO′) leads, where up-gaze gives a maximum response, significant changes in amplitude were also observed in electrodes remote from the eyes and in particular, from contralateral parietal-occipital (PO) and neck (CB′) leads. Short-latency potentials of similar latency were observed (p10/n17 and n10/p17, respectively). The pattern of change with gaze in the PO leads was distinct from that observed for the IO′ leads. For the PO leads, the maximum response was obtained with neutral gaze, and this was also distinct from that observed for CB′ electrodes, where a maximal response was observed with head flexion in the second wave but not the first. Evidence of modulation of N42 and N1 potentials with both eye and head position was also observed. Head- and eye-position manipulation thus suggests that the inion response consists of an early neurogenic component, as well as myogenic responses. The p10/n17 at PO, in particular, may be an indicator of vestibulocerebellar projections.

**NEW & NOTEWORTHY** Loud sounds were used to activate vestibular receptors in human volunteers and the effects of head and eye position studied for short-latency responses. A potential (p10/n17) recorded in the parieto-occipital leads showed behavior not expected for a response with a myogenic origin. Source modeling suggested a possible origin from the cerebellum. It may represent a new indicator of human vestibulocerebellar function.

## Abstract (precis)

Loud sounds were used to activate vestibular receptors in human volunteers and the effects of head and eye position studied for short-latency responses. A potential (p10/n17) recorded in the parieto-occipital leads showed behavior not expected for a response with a myogenic origin. Source modeling suggested a possible origin from the cerebellum. It may represent a new indicator of human vestibulocerebellar function.

since the work of Bickford et al. (1964), which established a vestibular-dependent and myogenic origin of the sound-evoked inion response, and subsequent work by Colebatch et al. (1994), the vestibular-evoked myogenic potential (VEMP) has been accepted as providing a reliable means for testing otolith function. In addition to the inion response-related VEMP of vestibulocollic origin, VEMPs may be recorded from periocular locations, and these are manifestations of vestibulo-ocular reflex (VOR) pathways (Rosengren et al. 2005; Todd et al. 2007). The vestibulocollic response recorded from over the sternocleidomastoid muscles (SCMs), the cervical VEMP has been established as being the result of inhibitory modulation of tonic electromyography (EMG). Its magnitude is therefore dependent on the state of contraction of the underlying SCM, either by rotation or extension of the head and neck (Colebatch et al. 1994; Colebatch and Rothwell 2004). The ocular VEMP (OVEMP) has similarly been established as having its origin in the modulation of activity in extraocular eye muscles (EOMs), specifically the inferior oblique and rectus, when recorded in infraocular locations (Weber et al. 2012).

Although the morphology of the OVEMP waveform is dependent on the specifics of the recording paradigm, i.e., the stimulus parameters and electrode placement (Chihara et al. 2007; Sandhu et al. 2013; Todd et al. 2007), a striking effect is that the waveform amplitude is largest when the subject's gaze is elevated (Govender et al. 2009; Rosengren et al. 2005). This has led to the proposal that this is due to variation with background EOM contraction by analogy to the cervical VEMP (Rosengren et al. 2005, 2013). However, this issue remains controversial, with at least two different (although not necessarily mutually exclusive) hypotheses proposed, i.e., proximity of the inferior oblique to the infraocular electrodes (Iwasaki et al. 2009) or central modulation of the otolith-ocular reflex by means of cerebellar influence on the vestibular nuclei (Todd et al. 2012).

As well as mediating the above reflexes, the vestibular system sends projections to the cerebellum (Barmack 2003; Carpenter et al. 1972; Wilson and Peterson 1978) and the cortex via several pathways (Lopez and Blanke 2011; Lopez et al. 2012). EEG recordings, following vestibular activation, yield vestibular-evoked potentials (VsEPs) of neurogenic origin, which reflect central, including cortical, processing of vestibular information (de Waele et al. 2001). The use of the same acoustic stimuli that produce VEMPs also gives rise to short-latency VsEPs when the intensity is above the VEMP threshold (Todd et al. 2003). Source analysis of the specific short-latency VsEP peaks, i.e., the centrally distributed P10 and the prefrontal/laterofrontal N15, which are coincident with the OVEMP n10/p17, shows that they also receive a contribution from ocular, as well as the neurogenic sources from central structures, including possibly the cerebellum or brain stem and frontal cortex (Todd et al. 2008). Other studies using alternative methods have also indicated a number of other possible cortical contributions to short-latency VsEPs produced by acoustic stimuli (McNerney et al. 2011).

It is possible that the central activity implicated by previous source analyses may be an index of central structures involved in the control or execution of the VOR. Such central activity might also, therefore, be modulated by gaze position, because gaze is known to modulate translational VOR gain (Angelaki 2004). Evidence of a gaze effect on potentials of central origin would throw light on the above issue of the origin of the gaze effect on the OVEMP. The aim of the present study was to investigate effect of gaze on short (and long)-latency VsEPs. To control for possible myogenic contributions from cervical muscles, we also included as a control the effects of head position.

## MATERIALS AND METHODS

### 

#### Subjects.

Eleven healthy subjects were recruited for this study (age range: 23–53; 7 women and 4 men). All subjects had no history of vestibular or hearing impairment. The experiment was conducted in two parts, and before testing, all subjects gave written consent, according to the Declaration of Helsinki. The study was approved by the Local South Eastern Sydney Local Health District Human Research Ethics Committee.

#### Stimuli.

Stimuli consisting of air-conducted (AC), 2 ms, 500 Hz, single-cycle tone pips, delivered by insert earphones (E-A-RTone Gold 3A; Guymark, West Midlands, UK), were used for VEMP and EEG recordings, fixed at a single intensity of +6 to 9 dB regarding 1 V peak [130–133 dB peak sound-pressure level (pk SPL)]. Stimulus calibration was carried out using a GRAS IEC711 Coupler (RA0045) and a pressure-field microphone (Model 4134) with a 2260 Investigator (Brüel and Kjaer, Naerum, Denmark). The stimuli were generated using customized software with a laboratory interface (Micro 1401; Cambridge Electronic Design, Cambridge, UK) and a custom amplifier.

#### Cervical VEMPs.

As only a single sound intensity was used in the present study, we did not measure exact vestibular thresholds but confirmed before recording EEG that cervical VEMPs were evoked. Subjects were tested lying supine on a couch, with the backrest tilted to ∼30–45° from the horizontal, and required to lift their heads against gravity to activate the SCMs. Surface EMG was measured from the ipsilateral SCM using self-adhesive Ag/AgCl electrodes. Active surface electrodes were placed over the middle of the SCM belly and were referred to electrodes placed on the medial clavicular head. EMG was amplified, bandpass filtered (5 Hz–1 kHz), and sampled using a Power1401 interface (Cambridge Electronic Design). The EMG was sampled at a rate of 5 kHz, starting 10 ms before to 80 ms following stimulus onset, and averaged. Stimuli were delivered by insert earphones (E-A-RTone Gold 3A; Guymark). Up to 200 stimuli were presented at a stimulus rate of ∼5 Hz. The presence or absence of a cervical VEMP was determined by visual inspection.

#### Vestibular-evoked potentials.

VsEPs were recorded with subjects seated upright and viewing a series of pictures at a distance of 1 m, which served to maintain concentration and minimize alpha waves. Subjects were asked to relax their facial muscles and remain alert. A neutral head position was maintained while EEGs were recorded during three different eye-gaze positions—neutral, eyes-up +20°, and eyes-down −20°—and then in two head positions—head-up +20° and head-down −20°—while maintaining a neutral gaze with respect to the head. The procedure was repeated for left- vs. right-ear stimulation, resulting in 10 recordings in total. We expected that VOR-related potentials would be modulated by eye position, whereas myogenic potentials arising from neck muscles would show modulation by head position. Stimulus presentation was randomized between 400 and 800 ms and set to a fixed intensity within the range given above. To avoid fatigue, recording was paused after 100 repetitions and resumed to obtain averages of ∼200 repetitions.

The EEG was recorded using a 64-channel EEG system (5.0; BioSemi, Amsterdam, Netherlands). Scalp electrode nomenclature largely follows the modified combinatorial nomenclature (Sharborough et al. 1991). Additional electrodes were also placed below each eye (i.e., infraocular electrodes, IO1′ and IO2′), at deep frontal (F9 and F10), at ear-lobe locations (A1 and A2), and over the splenius muscles of the neck (CB1′ and CB2′). Electrode offset (i.e., running average of the voltage measured between the common mode sense and each active electrode) was maintained below 20 μV. The common mode sense voltage also provided the average reference for the recording using all electrodes. Recordings were made with a band pass of between 0.16 Hz and 1 kHz, with sampling at 8,192 Hz. Artefact elimination, epoching, and averaging of evoked potentials (EPs) were carried out using Brain Electrical Source Analysis (BESA) 6 software (MEGIS Software, Gräfelfing, Germany). Epochs were 350 ms in length, from 50 ms before to 300 ms following the stimulus onset. After collection, EPs were filtered at 1–300 Hz and referenced to an average reference using Scan software (v4.3; NeuroScan, Charlotte, NC). The amplitudes and latencies of specific potentials of interest were measured in the ocular, central, and posterior electrodes for statistical analysis.

#### Source localization.

BESA software (6.0) was also used for source localization. The standard, four-shell, elliptical head approximation was used with the following parameters: the radial thickness of the head, scalp, bone, and cerebrospinal fluid was 85, 6, 7, and 1 mm, respectively, with conductivities set to 0.33, 0.33, 0.0042, and 1.0, respectively. As the principal effects we observed were for the short-latency waves, we focused our source analysis on the early part of the epoch (4–14 ms), for which purpose, the epoch was truncated to −20 to +100 ms, an additional high-pass filter of 5 Hz (0 phase shift, 24 dB/octave) was applied to remove low-frequency components, and a further correction was made to the baseline. In previous source modeling of short- and long-latency VsEPs, we have used symmetrical pairs of regional sources to capture the ocular contributions (Todd et al. 2008, 2014a, b). Given the large signal from IO′ leads, replicated in the present data, this approach seemed appropriate here. However, to avoid any bias in the initial source locations, we first applied simple dipole models using a genetic algorithm (one in which the best fitting solutions are used to generate the next iteration) to determine the likely principal contributions. Detailed analysis was initially conducted for the head-neutral, eyes-up condition, because this would allow the most accurate location of the ocular sources. Examination of the global field power (GFP) led us to define the analysis window to be 4–14 ms. This contained the first two lobes: a small, initial lobe with a peak at 6.9 ms and a second, larger lobe with a peak at 10.7 ms. The BESA genetic algorithm with default parameters was applied repeatedly over this window with models of successively increasing complexity. A second analysis was performed in which the two anterior periocular sources were made symmetrical, converted to regional sources, and the model refitted.

## RESULTS

### 

#### Grand means.

**Fig. 1.**
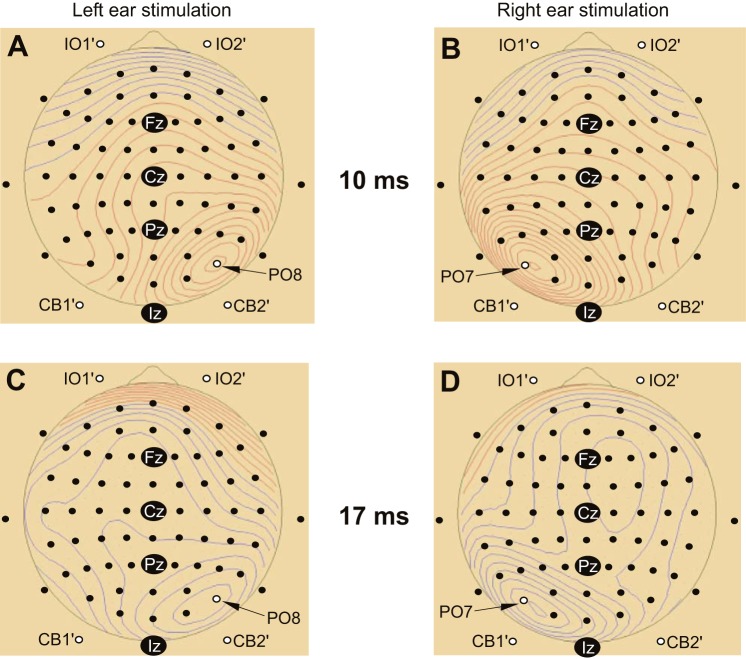
Top meridian-view scalp potential maps obtained from left-ear (*left*) vs. right-ear (*right*) stimulation in the neutral position at latencies of 10 ms (*A* and *B*) and 17 ms (*C* and *D*). At both latencies, there was a highly lateralized response that was focused in the contralateral PO8 and PO7 leads for left- and right-ear stimulation, respectively. Fz, Cz, Pz, and Iz refer to midline locations based on the 10–20 electrode system (Klem et al. 1999).

**Fig. 2.**
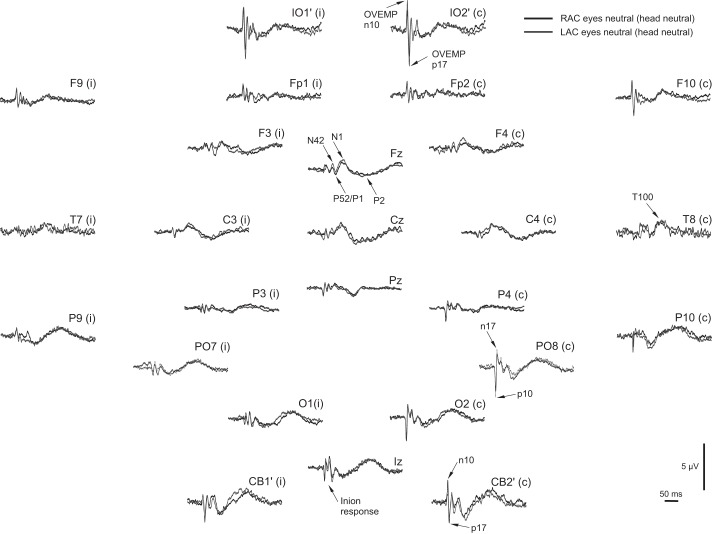
Array of grand means of evoked potentials elicited by AC sound at +6 to 9 dB regarding 1 V peak (130–133 dB pk SPL) for left (LAC; gray)- vs. right (RAC; black)-ear stimulation in neutral gaze. The right-ear grand mean has been reflected over the midline so that in the figure, the *left*/*right* side of the array represents responses ipsilateral (i)/contralateral (c) to the side of stimulation. Epoch is −50 to +300 ms.

**Fig. 3.**
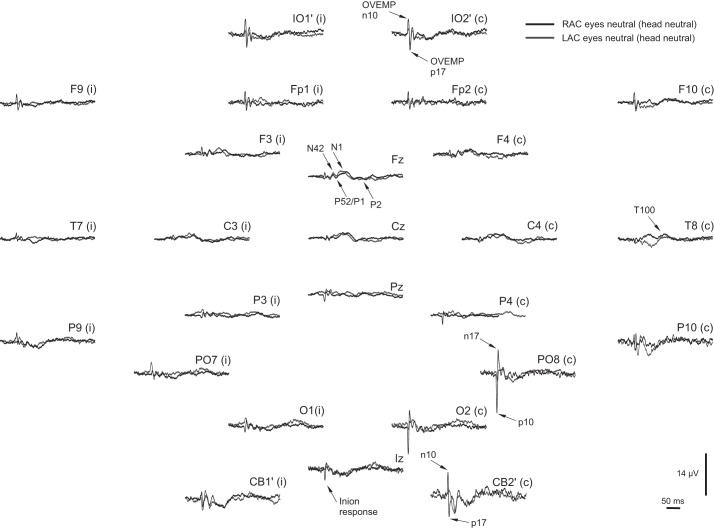
Array of evoked potentials from averaged EEG elicited by AC sound at +6 dB regarding 1 V peak (130 dB pk SPL) for left (LAS; gray)- vs. right (RAS; black)-ear stimulation in neutral gaze for a single representative subject. Individual trials (150–200; after artefact rejection) were used to construct each average trace and filtered 1–300 Hz. The right-ear grand mean has been reflected over the midline so that in the figure, the *left*/*right* side of the array represents responses ipsilateral (i)/contralateral (c) to the side of stimulation. Epoch is −50 to +300 ms.

The grand means of the average evoked EEG for the head-neutral/eye-neutral condition for left- and right-ear stimulation showed marked lateralization for the posterior group of electrodes, contralateral to the stimulus, and centered around parietal-occipital (PO)7 and PO8 for right- and left-ear stimulation, respectively (Fig. 1). One subject had excessive frontalis noise in some conditions and was excluded from all averaging and source analyses to avoid myogenic contamination but was retained in the ANOVAs, since accurate measurements could be made in the relevant electrodes. Another subject had corrupted or missing data in some conditions and was also excluded. Thus a total of *n* = 9 subjects were used for the figures and *n* = 10 for the ANOVAs. This did not invalidate the analyses, because the addition or subtraction of the one subject did not significantly change the morphology for the critical electrodes that were used in the statistics. Given the strong lateralization observed, we made grand averaged EPs to left- and right-ear stimulation but with the right-ear electrodes reflected over the midline (Figs. 2 and 3). For the remainder of the article, electrode locations are referred to as if the left ear was being stimulated. As expected from previous studies, the IO′ leads showed an initially negative, OVEMP-related response. For the posterior leads, most notably in contralateral PO leads, there were also short-latency responses but with an initial positivity. At FCz, there were N42, N1, and P2 waves. An inion-type response could also be seen in the Iz and the CB′ leads especially. These features in the grand mean could also be observed in individual subject data, as illustrated in Fig. 3.

#### Short-latency waves.

**Fig. 4.**
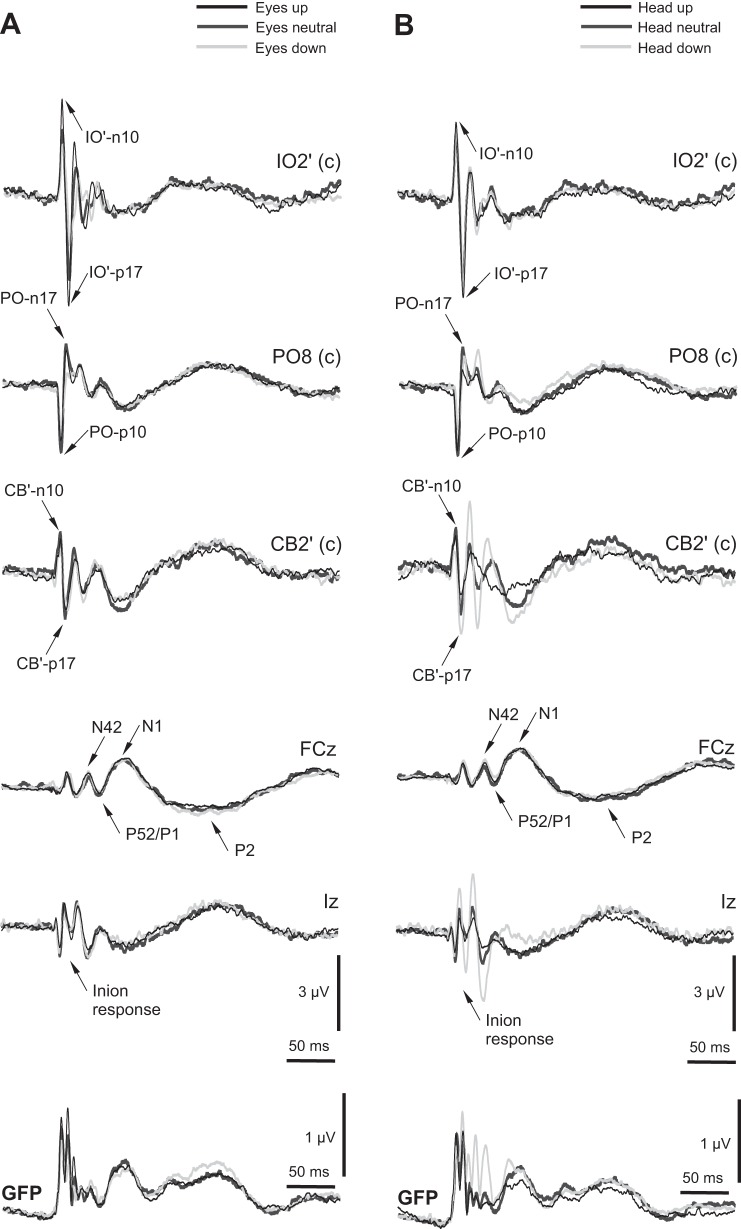
Grand means of evoked potentials from selected electrodes for 3 eye-gaze conditions of +20° up, neutral, and −20° down (*A*) and 3 head positions of +20° up, neutral, −20° down (*B*). The contralateral electrodes (IO2′, PO8, and CB2′) showed prominent short-latency response peaks. Midline electrodes (FCz and Iz) are also shown. The global field power (GFP) for the eye and head conditions are shown at the *bottom*.

In light of our initial assessment, we selected IO2′, FCz, PO8, Iz, and CB2′ for more detailed analysis. The OVEMP-related response in the IO′ lead consisted initially of n10/p17 potentials, which showed modulation with eye-gaze position (largest with up-gaze; Fig. 4*A*) but not with changes in head position (Fig. 4*B*). As noted, the PO lead demonstrated an early wave of similar latency but opposite polarity to the OVEMP, i.e., a p10/n17, and scalp maps from the grand average indicated that these were largest at PO8 (Fig. 1). Unlike the OVEMP response in IO leads, however, which showed eye position but no head position effects, the posterior leads showed different effects for both head and eye position. Both the Iz and CB′ leads were strongly modulated by head position, consistent with a cervical VEMP origin, particularly for potentials following the earliest one.

**Fig. 5.**
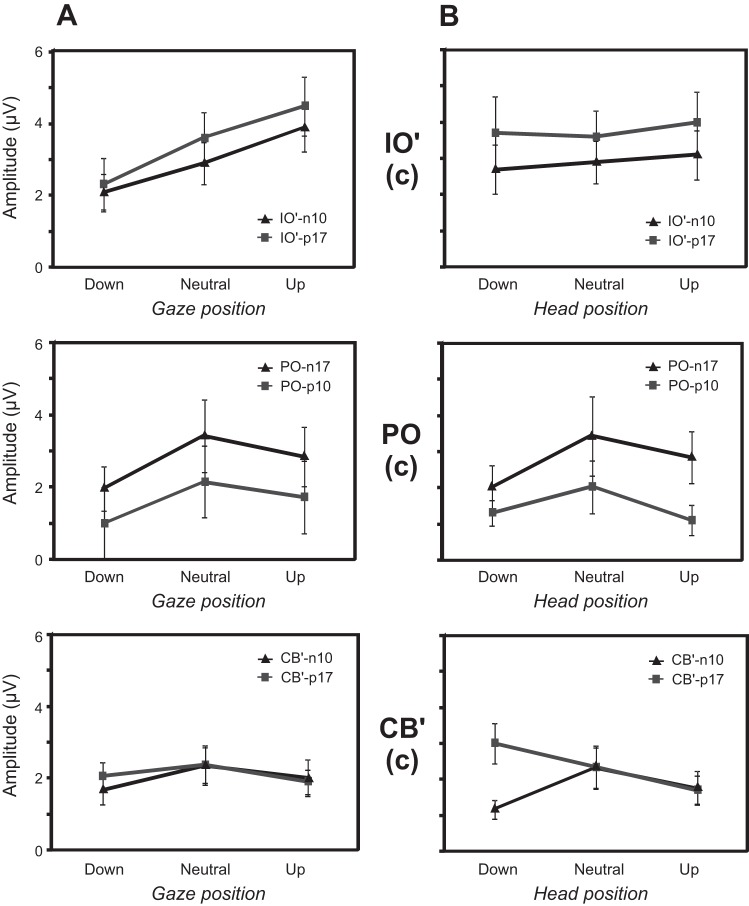
Estimated marginal means (i.e., corrected for covariates) of IO′ and CB′ n10/p17 and PO p10/n17 peaks as a function of eye gaze (*A*) and head (*B*) position. Estimated marginal means for the contralateral (c) IO′ and CB′ leads are shown at the *top* and *bottom*, respectively. *Middle*: contralateral PO7 or PO8 marginal means. Error bars represent the SE.

To investigate the apparent effects observed above within subjects, ANOVA was carried out for the different electrode sites for the initial biphasic potential amplitudes, using factors of eye or head position, wave, and ear. With the consideration of first, the effects of eye-position manipulation at the IO′ leads (Fig. 5*A*), a significant main effect of gaze was shown [F_(2,18)_ = 12.8, *P* < 0.001], with no other main effects. For head position (Fig. 5*B*), there was no main effect, although there was an effect of wave [F_(1,9)_ = 7.8, *P* < 0.05], where the n10 was different in size from the p17. Thus as expected, a change in eye-gaze position strongly modulated the magnitude of the contralateral IO′ amplitude, where up-gaze produced the largest response, but head-position change did not affect IO′ amplitude.

When the same analyses were applied to the PO lead, eye position also produced a significant main effect [F_(2, 18)_ = 3.6, *P* < 0.05], but here, the largest response was obtained with neutral gaze (Fig. 5*A*). A main effect of wave was also obtained [F_(1,9)_ = 5.9, *P* < 0.05], where the p10 was different in size from the n17. Whereas there was not a significant main effect of head position, there was a significant linear contrast for head position by wave [F_(1,9)_ = 5.8, *P* < 0.05]. However, as for eye gaze, the neutral head position produced the largest magnitude for both waves.

For the CB′ electrodes, there was no main effect of eye position (Fig. 5*A*), although the magnitude was largest with the eyes in a neutral position. However, for head position, although there was also no main effect, there was a highly significant interaction between head position and wave [F_(2, 18)_ = 13.0, *P* < 0.001], such that the second wave had a highest response in head-down position, in contrast to the first wave that was maximal in the neutral position (Fig. 5*B*). Again, there was a main effect of wave [F_(1,9)_ = 5.9, *P* < 0.05], but in this case, the main effect was probably a result of the amplitude difference in the head-down position between the two waves. As there appeared to be a clear difference in behavior between the two CB′ waves, we conducted a further ANOVA using the magnitude of the first waves at the PO7 and PO8 (p10) and CB1′ and CB2′ (n1) locations. For eye-position manipulation, the inverted V shape was shown at both electrodes but did not reach significance, whereas for head position, the inverted V-shape effect was significant [F_(2, 18)_ = 4.2, *P* < 0.05], with also a main effect of electrode [F_(1,9)_ = 5.3, *P* < 0.05].

Although the behavior of the magnitudes across the IO′, PO, and CB′ electrodes was different with manipulation of eye and head position, of interest also was the comparison across individuals of the magnitude of each of the responses in the neutral position (see Table 1). A correlation of magnitudes for the initial waves in each of the three electrode positions (contralateral IO′, PO, CB′) showed that for the same ear of stimulation, there were indeed high within-ear correlations (from *r* = 0.86 to 0.97) but not between ears (from *r* = −0.17 to 0.11), i.e., so that each ear could be treated as a different case.

**Table 1.** Correlation matrix for 10 ms waves measured at contralateral IO′, PO, and CB′ leads in the neutral position (n = 11)

		L IO′	L PO	L CB′	R IO′	R PO	R CB′
L IO′	*r*						
	*P*						
L PO	*r*	0.871					
	*P*	**<0.001**					
L CB′	*r*	0.868	0.964				
	*P*	**0.001**	**<0.001**				
R IO′	*r*	0.110	0.021	−0.079			
	*P*	0.747	0.951	0.817			
R PO	*r*	−0.003	0.038	−0.133	0.843		
	*P*	0.994	0.912	0.697	**0.001**		
R CB′	*r*	−0.004	−0.104	−0.172	0.927	0.874	
	*P*	0.990	0.760	0.613	**<0.001**	**<0.001**	

L, left; R, right. Boldface refers to significant *P* values.

#### Mid- and long-latency waves.

**Fig. 6.**
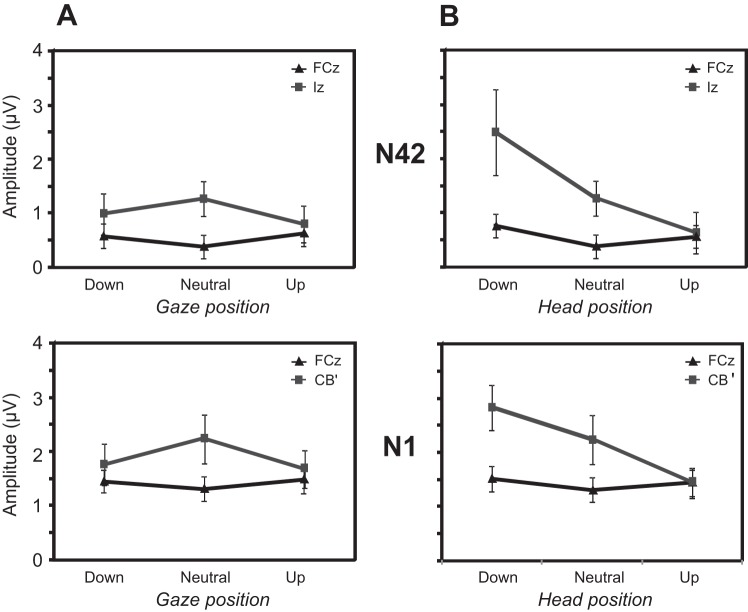
Estimated marginal means of N42 and N1 peaks as a function of eye gaze (*A*) and head (*B*) position. *Top*: N42 marginal means measured at FCz and Iz leads; *bottom*: N1 marginal means at FCz and CB. Error bars represent the SE.

In addition to the short-latency effects, we investigated the effects of head and eye position on longer-latency waves (Fig. 6). N42 wave measurements were taken at FCz and Iz, since a scalp analysis showed that maxima of this potential and its inverse were located at these two electrodes. For the N42, there was no main effect of eye position, although there was a significant interaction between position and electrode [F_(2, 16)_ = 7.4, *P* < 0.01], whereby at Iz, the N42 was maximal with eyes neutral, and at FCz, the opposite pattern was observed (Fig. 6*A*). For head-position changes, there was a main effect [F_(2, 16)_ = 5.8, *P* < 0.05] and also an interaction between position and electrode [F_(2, 16)_ = 5.2, *P* < 0.05], where for Iz, the magnitude was greatest with head down, whereas for FCz, the N42 peak was again smallest with the head neutral (Fig. 6*B*). When the same analyses were conducted for the N1 response with measurements at FCz and the contralateral CB′ electrodes, the pattern of results was similar to the N42 (Fig. 6). So for eye-position manipulation, the interaction effect showed the same inversion between FCz and the posterior electrode responses [F_(2, 16)_ = 3.8, *P* < 0.05]. For head-position manipulation, a main effect [F_(2, 16)_ = 7.5, *P* < 0.005] and an interaction between position and electrode were obtained [F_(2, 16)_ = 5.7, *P* < 0.05].

#### Source analyses.

**Fig. 7.**
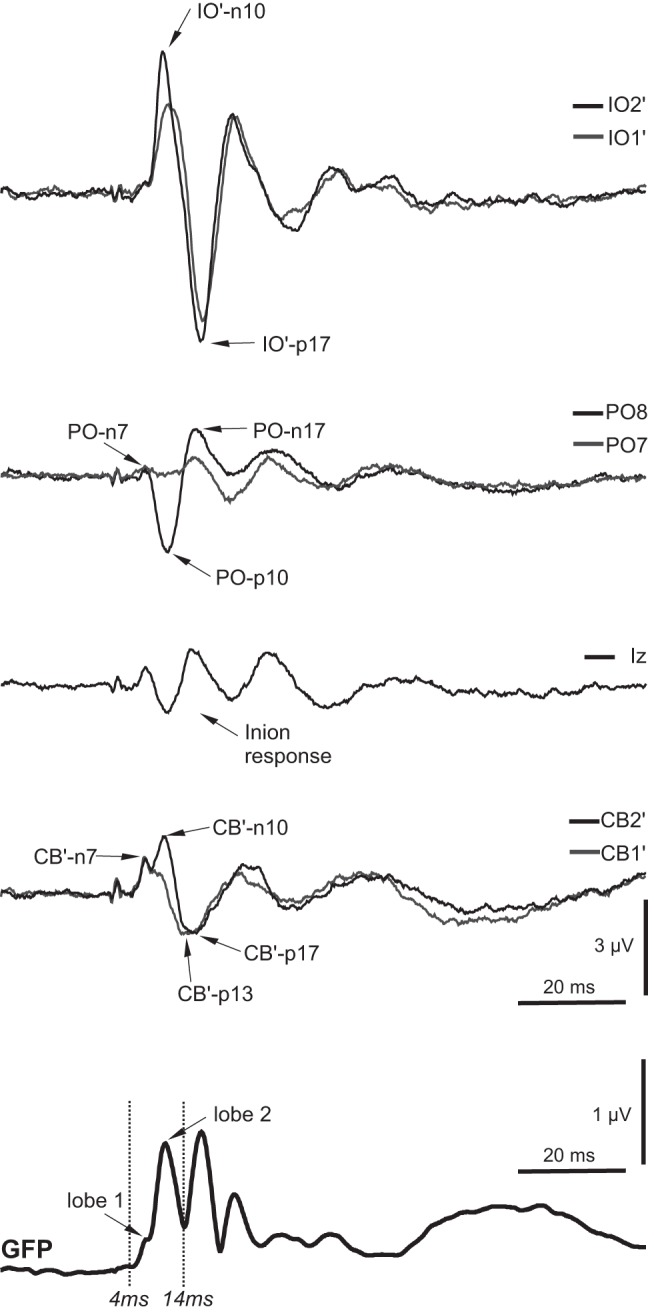
Grand means of evoked potentials for selected leads along with the global field power (GFP) for the up-gaze condition after reducing the epoch to −20 to +100 ms and high-pass filtering at 5 Hz. The source fitting corresponded to lobes 1 and 2 of the GFP.

As noted above, the source analysis was restricted to the early part of the epoch (4–14 ms), for which purpose, the epoch was truncated to −20 to +100 ms (Fig. 7). In previous source modeling of short- and long-latency VsEPs, we have used symmetrical pairs of regional sources to capture the ocular contributions (Todd et al. 2008, 2014a, b). Given the large signal from IO′ leads, replicated in the present data, this approach seemed appropriate here. However, to avoid any bias in the initial source locations, we first applied simple dipole models using a genetic algorithm to determine the likely principal contributions.

For models with one, two, or three dipoles, unique solutions were obtained. With the three-dipole model, already 94% of the variance could be explained by two anterior sources: one located to the contralateral orbit and a contralateral posterior source that located to the cerebellar hemisphere. The second anterior source localized near the midline between the orbits, suggesting a bilateral ocular source in addition to the contralateral ocular source. For models with four or more dipoles, there were multiple possible solutions, the most common of which are summarized in Table 2. Extraocular and contralateral cerebellar sources were present for all three and greater dipole models. The higher-order models indicated that in addition to the principal ocular and contralateral cerebellar sources, there were smaller contributions from the ipsilateral cerebellum and from the cervical muscles, as well as from the frontal cortex.

**Table 2.** Successive simple dipole models

Model	X, Y, Z	Antero-Postero Location	RV, %	Possible Origin
1 DP	28, −8, −46	Post	23.4	
2 DP	19, 26, −48	Ant	12.9	
	41, −65, −57	Post		
3 DP	19, 66, −25	Ant	5.8	Contra EOMs
	−3, 41, −47			EOMs
	35, −73, −45	Post		Contra cerebellum, inf. semi-lun. l.
4 DP	23, 71, −32	Ant	3.6	Contra EOMs
	−5, 36, −26			EOMs
	35, −77, −46	Post		Contra cerebellum, inf. semi-lun. l.
	−16, −45, −56			Ipsi cerebellum/brain stem
4 DP 2	19, 72, −22	Ant		Contra EOMs
	−4, 35, −47			EOMs
	41, −78, −51	Post		Contra cerebellum, inf. semi-lun. l./cervical muscles
	−25, −58, −6			Ipsi occipital/temporal lobe/BA19
5 DP	21, 74, −27	Ant	2.5	Contra EOMs
	−5, 29, −38			EOMs
	37, −80, −49	Post		Contra cerebellum, inf. semi-lun. l./cervical muscles
	−23, −47, −38			Ipsi cerebellum, tonsil
	−31, −85, −53			Ipsi cervical muscles
6 DP	23, 72, −29	Ant	2.2	Contra EOMs
	−7, 46, −43			EOMs
	30, 30, −8			IFG/MFG/BA47, -11
	39, −80, −50	Post		Contra cerebellum, inf. semi-lun. l./cervical muscles
	−30, −47, −33			Ipsi cerebellum, tonsil
	−32, −85, −52			Ipsi cervical muscles
6 DP 2	19, 73, −29	Ant	2.2	Contra EOMs
	−1, 46, −39			EOMs
	52, 9, −12			STG/MTG/BA38, -21
	39, −80, −50	Post		Contra cerebellum, inf. semi-lun. l./cervical muscles
	−33, −52, −33			Ipsi cerebellum, tonsil
	−35, −82, −52			Ipsi cervical muscles
6 DP 3	18, 73, −32	Ant	2.4	Contra EOMs
	−7, 41, −32			EOMs
	21, 14, −13			IFG/insula/BA47, -13, putamen
	31, −85, −52	Post		Contra cerebellum, inf. semi-lun. l./cervical muscles
	−28, −50, −36			Ipsi cerebellum, tonsil
	−17, −70, −57			Ipsi cervical muscles
6 DP 4	18, 73, −32	Ant	2.4	Contra EOMs
	−5, 34, −33			EOMs
	38, −80, −50	Post	2.0	Contra cerebellum, inf. semi-lun. l./cervical muscles
	68, −27, −39			Contra postauricular muscle
	−32, −50, −37			Ipsi cerebellum, tonsil
	−29, −84, −54			Ipsi cervical muscles
7 DP	18, 73, −33	Ant	1.6	Contra EOMs
	0, 42, −41			EOMs
	43, 12, −10			STG/IFG/insula/BA38, -47, -13
	31, −85, −52	Post		Contra cerebellum, inf. semi-lun. l./cervical muscles
	41, −60, −19			Contra cerebellum, declive, tuber
	−28, −50, −36			Ipsi cerebellum, tonsil
	−17, −70, −57			Ipsi cervical muscles
7 DP 2	13, 70, −32	Ant	1.7	Contra EOMs
	−16, 48, −23			Ipsi EOMs
	26, 51, −12			SFG/MFG/BA11, -10
	39, −80, −51	Post		Contra cerebellum, inf. semi-lun. l./cervical muscles
	67, −24, −37			Contra postauricular muscle
	−26, −45, −32			Ipsi cerebellum, tonsil, tuber
	−32, −85, −51			Ipsi cervical muscles

Head-neutral, eyes-up condition. Successive dipole source models for the period 4–14 ms poststimulus. Suffixes 2, 3, and 4, multiple stable models. X, Y, and Z, stereotactic coordinates (Talairach and Tournoux 1988). RV, residual variance; DP, dipoles; Post, posterior; Ant, anterior; inf. semi-lun. l., inferior semi-lunar lobule; BA, Brodmann areas; IFG, inferior frontal gyrus; MFG, middle frontal gyrus; STG, superior temporal gyrus; MTG, middle temporal gyrus; SFG, superior frontal gyrus.

**Fig. 8.**
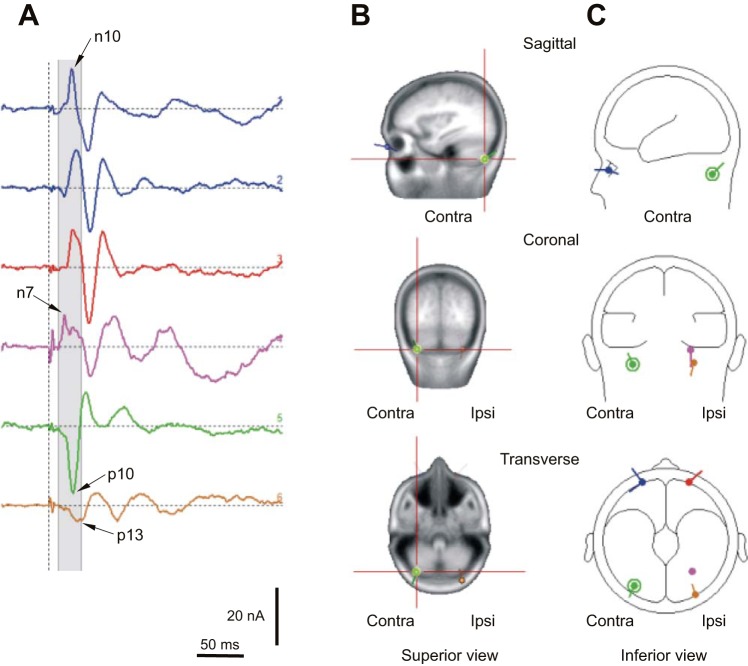
Model with a pair of ocular sources and 3 dipoles. *A*: the source waveforms: source 1 (first blue trace), contralateral tangential ocular; source 2 (second blue trace), contralateral radial ocular; source 3 (red trace), ipsilateral radial ocular; source 4 (purple), ipsilateral cerebellar; source 5 (green trace), contralateral cerebellar; and source 6 (brown trace), ipsilateral cervical. Source locations are shown using standard MRI (*B*) and BESA (*C*) images, with source 5 highlighted. The gray zone is the period on which the source fitting was based.

With the use of the simple dipole models as the starting point, the two anterior sources were made symmetrical, converted to regional sources, and the model refitted. The outcomes of this approach are summarized in Table 3. For models with the ocular pair plus one or two dipole sources, the solutions were unique and again, indicated a large, contralateral cerebellar source located in the hemisphere and a smaller, more medial ipsilateral cerebellar source located within the hemisphere or the tonsil. With the pair plus three dipoles, there were two stable solutions, both of which included bilateral cerebellar sources and an extracranial source within the ipsilateral cervical region. Both of these solutions had low residual variance (2.1% and 2.2%). With an additional dipole, a contralateral cervical source was present, but there was no further reduction in residual variance. Therefore, we preferred the three-dipole solution with the lower residual variance, as shown in Fig. 8.

**Table 3.** Ocular regional sources plus additional dipole models

Model	X, Y, Z	Antero-Postero Location	RV	Possible Origin
Pair + 1 DP	±17, 64, −28	Ant	4.8%	Bilateral EOMs
	35, −72, −46	Post		Contra cerebellum, inf. semi-lun. l.
Pair + 2 DP	±25, 73, −26	Ant	3.5%	Bilateral EOMs
	34, −72, −46	Post		Contra cerebellum, inf. semi-lun. l.
	−16, −45, −56			Ipsi cerebellum, inf. semi-lun. l., tonsil
**Pair + 3 DP**	**±26, 70, −31**	**Ant**	**2.1%**	**Bilateral EOMs**
	**35, −75, −49**	**Post**		**Contra cerebellum, inf. semi-lun. l./cervical muscles**
	**−27, −57, −34**			**Ipsi cerebellum, tonsil**
	**−35, −86, −49**			**Ipsi cervical muscles**
Pair + 3 DP 2	±26, 70, −31	Ant	2.2%	Bilateral EOMs
	40, −79, −51	Post		Contra cerebellum, inf. semi-lun. l./cervical muscles
	−16, −54, −16			Ipsi cerebellum, culmen, declive, dentate
	−32, −86, −50			Ipsi cervical muscles
Pair + 4 DP	±26, 70, −32	Ant	2.2%	Bilateral EOMs
	36, −72, −48	Post		Contra cerebellum, inf. semi-lun. l.
	15, 79, −23			Contra cervical muscles
	−18, −87, −57			Ipsi cervical muscles
	−38, −52, −27			Ipsi cerebellum, culmen, tonsil, tuber
Pair + 4 DP 2	±26, 71, −31	Ant	2.2%	Bilateral EOMs
	55, −58, −35	Post		Contra cerebellum, tonsil, inf. semi-lun. l.
	35, −78, −55			Contra cervical muscles
	−22, −86, −57			Ipsi cervical muscles
	−27, −56, −28			Ipsi cerebellum, culmen, tonsil

Results obtained using 2 anterior regional ocular sources and then adding dipole sources. Suffix 2, additional solution. X, Y, and Z, stereotactic coordinates (Talairach and Tournoux 1988). RV, residual variance; DP, dipoles; Post, postero; Ant, antero; inf. semi-lun. l., inferior semi-lunar lobule. Boldface indicates the solution illustrated in Fig. 8.

The ocular regional sources were rotated so that their first component was maximal and then the individual dipole components displayed in an attempt to determine the major dipole contributions and thereby reduce the number of components. On the side contralateral to the stimulus, there were two significant components within the fitting window: one radial and one tangential to the eye, with a single radial component on the ipsilateral side. For the illustration, the other, lesser components were deleted. The contralateral tangential component (source 1; Fig. 8*A*) peaked at 9.8 ms, corresponding to the n10 OVEMP in IO′ leads. The bilateral radial components (sources 2 and 3) looked similar and had peak amplitudes at 11.8 ms. Before these peaks, the ipsilateral cerebellar source (source 4) had a peak at 6.7 ms, corresponding to the first small lobe in the GFP (Fig. 7) and the CB′–n7 wave (Fig. 7). This was followed by a peak for the contralateral cerebellar source (source 5) at 10.1 ms, which corresponded with the p10 wave in PO leads. The last source, located within the ipsilateral cervical region, had an initial peak at ∼13 ms.

**Fig. 9.**
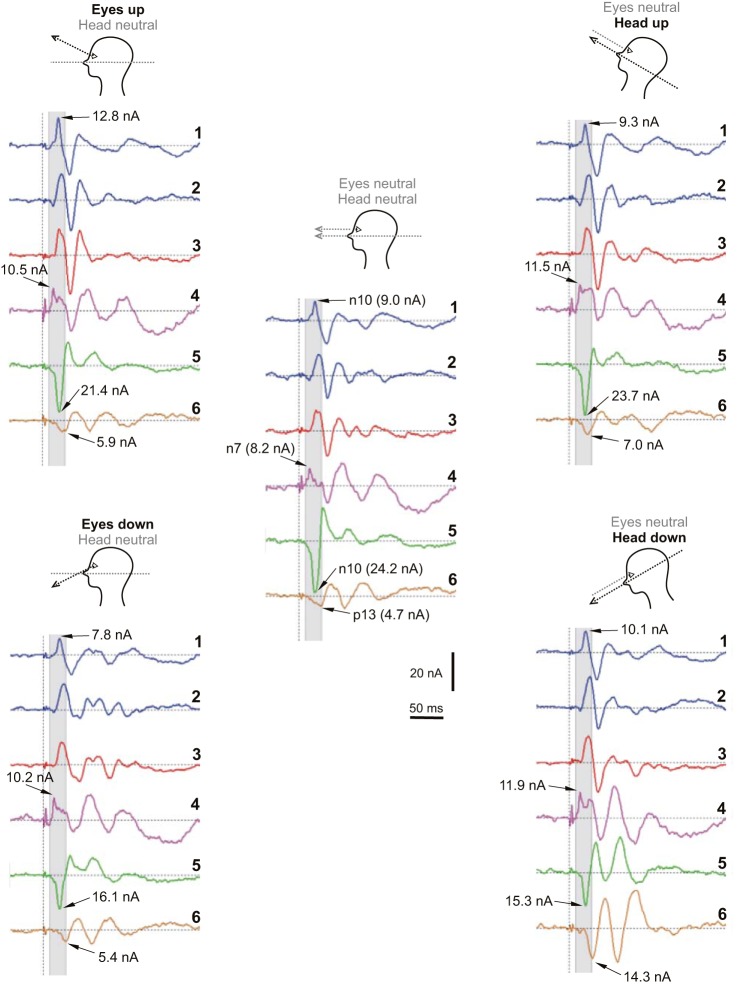
The selected model response for each of the 4 eye and head positions. *Left*: up vs. down eye position. *Right*: up vs. down head position. Source 1 (first blue trace), contralateral tangential ocular source (and likely OVEMP n10 generator), at 10 ms is largest in up gaze and smallest in down gaze. Source 4 (purple trace), ipsilateral cerebellar source (and likely CB′–n7 generator), is smallest in the neutral position. Source 5 (green trace), contralateral cerebellar source (and likely PO–p10 generator), is largest in the neutral position. Source 6 (brown trace), ipsilateral cervical source (and primary posterior cervical VEMP–p13 generator), is largest in head-down position.

In Fig. 9, we show how the sources alter with the changes of eye and head position. With eye-position alterations, the contralateral tangential ocular n10 (source 1) was largest in the up-gaze position (12.8 vs. 9.0 and 7.8 nA for neutral and down). In contrast, the contralateral cerebellar p10 (source 5) was largest in the neutral position (24.2 vs. 21.4 and 16.1 nA for eyes-up and -down cases). The ipsilateral cervical p13 (source 6) changed little with eye position, with values of 5.4, 4.7, and 5.4 nA for the up, neutral, and down positions, respectively. For head-position changes for the ocular sources, the contralateral tangential ocular n10 source did not change significantly, remaining close to the neutral value of 9.0 nA, but the ipsilateral cervical source increased to 14.3 nA for the head-down case. As for the eye-gaze changes, the contralateral cerebellar p10 current (source 5) was largest in the neutral position, with values of 23.7 and 15.3 nA for head-up and -down cases. The ipsilateral cerebellar n7 (source 4) did not show a large change across conditions but was smallest in the neutral case, with a value of 8.2 nA and corresponding values of 10.5 and 10.2 nA for eyes-up and -down cases and 11.5 and 11.9 nA for head-up and -down cases.

## DISCUSSION

The data presented here used intense sound to activate the vestibular afferents, and this appears to be selective for irregular afferents from both otoliths (Curthoys et al. 2006). We have replicated previous results showing that the magnitude of vestibular-dependent potentials recorded in periocular locations is strongly influenced by gaze position (Govender et al. 2009; Rosengren et al. 2005). Our results also provide new findings for what appear to be vestibular-dependent potentials recorded in locations remote from the eyes, which are also affected by gaze and head position. Analysis of the n10/p17 waves in IO1′ and IO2′ confirmed prior experimental results that for AC stimuli, the contralateral n10/p17 waves, in particular, were strongly dependent on gaze position, with the largest amplitude obtained with upward gaze (Govender et al. 2009). Whereas in the present case, we used a referential montage rather than the usual differential one so that nonlocal sources are also likely to contribute, the results we obtained are similar to that of Govender et al. (2009).

In the present study, we recorded additional short-latency potentials of opposite polarity (p10/n17) in the contralateral PO leads, as well as at the inion (Iz) and contralateral CB′ leads. We have used the modified combinatorial nomenclature to locate our cortical electrode positions (Sharbrough et al. 1991), and in this arrangement, most cortical electrodes are separated by 10% of the relevant circumference or coronal section. In particular, PO7 and PO8 lie on the major circumference, including Fpz and Oz, the latter lying in the midline at 10% and 90% of the distance between the nasion and the inion. PO7 and PO8 lie 20% of the hemicircumference to the left and right of Oz, respectively. Given that they lie at the same level as Oz, PO7 and PO8 are likely to lie over the occipital lobes, above the anterior lobe of the cerebellum. Although the PO p10/n17 shared the property with the IO n10/p17—being strongly lateralized to the contralateral side—the response was dissociated from the IO′ ones in the way that they were modulated by eye position, whereby the largest response for both was obtained in the neutral position. In contrast to the IO′ n10/p17, the PO p10/n17 appeared to be modulated by head position. This is unlikely to be explained by the presence of a contribution from the inion response, because the behavior was dissociated from that of waves of similar latency in the modified CB′ electrodes placed over splenius muscles. The CB′ waves were not strongly modulated by eye-gaze position, but there was a strong interaction between wave and head position, such that the second wave (p17) at CB′ behaved as if it included a component of the inion myogenic response. In the head-down position, there will be an increased contraction of neck extensors to hold the head against the force of gravity, and so we would expect that a cervical VEMP component arising from the neck extensors would be largest with head down. This behavior was not observed for either of the PO waves. The failure to change with head position and thus the apparent absence of a myogenic contribution to the initial wave in the CB′ leads (n10) suggest that it may be related to the wave of similar latency but opposite polarity in the PO leads.

With the analysis of waves of longer latency, the N42 and N1 indicated a difference between the anterior and posterior components. Magnitudes of the N42 and N1 at FCz and in Iz or contralateral CB′ were modulated in the opposite direction, such that at FCz, the N42 and N1 waves were smallest in the neutral eye position, in contrast to the largest response obtained in the neutral position for the posterior measurements. For head-position changes, the posterior measurements showed the response that one would expect if there was a myogenic contribution from the inion response. This, however, could not explain the apparent effect of eye-gaze position on the longer latency waves, since the head remained in the neutral position, and thus there should have been no change in cervical muscle contraction. The dissociation that we observed for the short-latency PO p10/n17 and the CB′ n10 from both the OVEMP and cervical VEMP behavior and the additional evidence of modulation of longer-latency waves with eye position suggest that the origin of change in magnitude in these leads with gaze may include a central component. The PO leads lie over the skull, with little muscle underlying them. Across individuals and ears, there was a strong correlation in the overall size of the responses so that although they were modulated differently by eye gaze and head position, they are likely to represent different responses to the same peripheral input.

We used BESA to model our sources, an approach that is based on using discrete dipole sources and is one of a number of methods to solve the “inverse problem” of deriving sources from surface EEG (Grech et al. 2008; Michel et al. 2004). BESA has the specific advantage for our studies in that it allows for non-neural and extracranial sources, an essential feature for modeling the earliest events as we have here, given the strength of the OVEMPs. Miltner et al. (1994), who assessed an earlier version of the BESA software, point out that six noiseless electrode recordings are required to derive the properties of a single dipole, and in the presence of noise, a unique solution may not be possible. The use of an elliptical head-model approximation, as adopted here, will also limit the accuracy of source localization. In the simulation by Miltner et al. (1994), using 32 electrodes, 7 of the 10 sources were usually detected with an overall mean average error of 1.4 cm. It was also clear that solutions based on awareness of the underlying physiology were more successful than those without. Consistent with this, we, as previously, fixed two regional sources to correspond with the known origins of OVEMPs. We have also used a higher ratio of electrodes to fitted sources than Miltner et al. (1994) and have fitted during a high signal-to-noise part of the waveform, consisting of lobes one and two of the GFP (Fig. 7).

The source models consistently located bilateral ocular sources with a contralateral tangential source, in particular, corresponding to the contralateral n10 OVEMP generator. At the same latency, a large contralateral source was consistently located using both the three- and four-dipole models to the cerebellar hemisphere. The size of this potential correlated with the size of the known vestibular-dependent OVEMPs. We suggest therefore that the contralateral cerebellum is a plausible candidate for a central generator of the PO–p10 and CB′–n10. Our present source model is also consistent with our earlier modeling work. Todd et al. (2008) conducted a source analysis of short-latency VsEPs evoked by both AC and bone-conducted vestibular stimulation. They concluded that in addition to bilateral ocular sources, there must be deep sources coincident with the EOM sources, which they suggested further were likely to correspond to the cerebellum or brain stem, in addition to smaller frontal sources. More recent source analyses of short- and long-latency VsEPs have also consistently indicated the presence of cerebellar sources that appear to be lateralized and with an ear advantage (Todd et al. 2014a, b), although cerebellar activation has not been a universal finding (McNerney et al. 2011). The cerebellar sources also appear to show some sensitivity to stimulus rate, consistent with the role of the cerebellum in stabilizing gaze and posture during locomotion (Todd et al. 2016).

It is well established that the cerebellum plays a central role in the control of gain and phase of the VOR and of smooth-pursuit eye movements (Ito 1982). In the case of otolith-ocular reflexes (mediated by a 3-neuron arc through vestibular and oculomotor nuclei following afferent input from the otolith organs), central gain control is thought to be achieved primarily by the nodular/uvular and floccular divisions of the cerebellum, which receive mossy fiber primary-afferent input from the vestibular apparatus and secondary projections from the vestibular nuclei (Barmack 2003; Büttner-Ennever 1999; Voogd et al. 2012; Walker et al. 2010). The nodulus/uvula and floccular lobes exert their influence by means of Purkinje cell inhibition of the vestibular nuclei, but the strength of the influence can be modulated by retinal and eye proprioceptive inputs to the cerebellum via the inferior olive and climbing fiber inputs to the nodulus/uvula and floccular lobes (Barmack 2003; Voogd et al. 2012). The gaze effects that we have observed in the p10 in posterior leads could therefore represent a modulation of activity of the vestibular cerebellum with changing positions of the eyes. The fact that the largest response that we observed in the PO–p10 and in the corresponding contralateral cerebellar source was found in the neutral position is consistent with it representing inhibitory Purkinje cell activity. VOR gain is required to be larger in more eccentric gaze positions (Angelaki 2004); hence, inhibitory gain control is required to be lower.

We also identified an earlier ipsilateral cerebellar source with a peak activity at 6.7 ms (n7). It may correspond to the n6 potential of Papathanasiou et al. (2010), who reported its presence in posterior electrodes ipsilateral to the ear stimulated. They showed that the potential had a high threshold and did not depend on hearing but proposed that it was likely to have a brain stem origin. Unlike the contralateral ocular or contralateral cerebellar sources, the ipsilateral cerebellar n7 potential did not change greatly with head or eye manipulations, although it was smallest in the neutral position. The ipsilateral cerebellar source was generally placed near the cerebellar tonsil, which corresponds to the paraflocculus, a part of the floccular lobe. This early peak may thus represent the primary vestibular afferent activity known to project to the vestibular cerebellum as mossy fibers, most strongly to the nodulus/uvula (Barmack 2003; Carpenter et al. 1972; Voogd et al. 2012). The later, 10-ms contralateral cerebellar source activity may represent a climbing fiber input and Purkinje cell discharges related to complex gain control of the oculomotor system (Voogd et al. 2012). The source localization mainly placed the contralateral source in the cerebellar hemisphere, which is considered neither primary nor secondary vestibular areas. The inferior semilunar lobule is, however, established as an oculomotor area (Voogd et al. 2012), and during optokinetic activation, the cerebellar hemispheres are strongly activated (Bense et al. 2006). Whereas this interpretation assumes that vestibular afferents activated by sound are responsible, acoustic responses have been shown for single-unit recordings in the vermis, which are likely to be cochlea dependent (Aitkin and Boyd 1975; Altman et al. 1976). The lateralization of our findings and the correlation with OVEMP amplitudes favor a vestibular source, but some caution at this stage is required in concluding that this is the case.

Todd et al. (2012) reported evidence that changes in the OVEMP gain with varying gaze were itself both context and frequency dependent, effects that could not be explained by peripheral EOM origins alone. The present data provide further evidence that the gaze effects observed in IO′ leads may also have a central component. For an EEG montage with linked ears or an average reference, at least some contribution to periocular potentials must be due to remote sources that are coincident with the EOM generators. The use of the differential OVEMP montage increases selectivity to local, presumably EOM sources (Govender et al. 2016), yet the patterns observed here for changes in OVEMP amplitudes with gaze (Fig. 4) are qualitatively similar to those of Govender et al. (2009). Thus it is possible that the differential montage cannot completely exclude a remote contribution. The difference in behavior of the contralateral IO′ n10 from the PO p10 does suggest, however, that the n10 component, at least, is primarily local and EOM in origin, as is generally believed (Weber et al. 2012). Our observations and associated source models should also prompt a reappraisal of the inion response. Whereas it has been generally assumed since the work of Bickford et al. (1964) that the inion response was entirely myogenic in origin, the evidence presented here suggests that central, likely cerebellar, sources also contribute to the early and possibly later components. The PO p10/n17 complex may have a role in assessing vestibulocerebellar function in human subjects.

## GRANTS

Support for the research reported in this article was partly provided by a grant from the Wellcome Trust (WT091961MA) held at the University of Manchester, UK.

## DISCLOSURES

No conflicts of interest, financial or otherwise, are declared by the authors.

## AUTHOR CONTRIBUTIONS

N.P.M.T., S.G., and J.G.C. performed experiments; N.P.M.T., S.G., and J.G.C. analyzed data; N.P.M.T., S.G., and J.G.C. interpreted results of experiments; N.P.M.T. and S.G. prepared figures; N.P.M.T. drafted manuscript; N.P.M.T., S.G., and J.G.C. edited and revised manuscript; N.P.M.T., S.G., and J.G.C. approved final version of manuscript.
